# Complete mitogenomes of the chlorophyte green algae *Scherffelia dubia* and *Tetraselmis* sp. CCMP 881 (Chlorodendrophyceae)

**DOI:** 10.1080/23802359.2019.1698349

**Published:** 2020-01-08

**Authors:** Monique Turmel, Christian Otis, Jean-Charles de Cambiaire, Claude Lemieux

**Affiliations:** Département de biochimie, de microbiologie et de bio-informatique, Institut de Biologie Intégrative et des Systèmes, Université Laval, Québec, Canada

**Keywords:** Chlorophyta, Chlorodendrophyceae, inverted repeat, mitogenome evolution, phylogenomics

## Abstract

We report here the first mitogenome sequences for the chlorophyte class Chlorodendrophyceae. The mitogenomes of *Tetraselmis* sp. CCMP 881 and *Scherffelia dubia* (SAG 17.86) are 46,904 bp and 78,958 bp long, respectively, but their gene repertoires are almost identical. Each genome harbors an inverted repeat (IR). The 14,105-bp IR of *S*. *dubia* encodes seven genes in addition to a part of *rps19*, whereas the 2445-bp IR of *Tetraselmis* sp. CCMP 881 contains a single gene. Considering that an IR has also been found in the mitogenomes of certain earlier-diverging chlorophytes, the IRs of chlorodendrophycean algae probably represent ancestral features.

The Chlorodendrophyceae is a small class of green algae belonging to the Chlorophyta that comprises marine and freshwater scaly quadriflagellates (Massjuk 2006; Leliaert et al. 2012). In this study, we sequenced the mitogenomes of *Tetraselmis* sp. CCMP 881 and *Scherffelia dubia* (Chlorodendrales, Chlorodendraceae). These sequences are the first complete mitogenomes available for the Chlorodendrophyceae.

The *S. dubia* strain (SAG 17.86) was obtained from the Culture Collection of Algae at the University of Göttingen, whereas the *Tetraselmis* sp. strain (CCMP 881) was obtained from the Bigelow National Center for Marine Algae and Microbiota (Maine, USA). For DNA isolation, DNA sequencing and gene annotation, we used the methodologies we previously described for the plastomes of the same algal strains (Turmel et al. 2016). Briefly, random clone plasmid libraries were prepared from an A + T-rich DNA fraction and inserts of selected clones were sequenced using the Sanger method. Genomic regions, not represented in the clones analyzed, were sequenced from PCR fragments. All sequence data were generated by the “Plateforme d’Analyses Génomiques” of Laval University and they were edited and assembled using Sequencher 5.1 (Gene Codes Corporation, Ann Arbor, MI).

The *Tetraselmis* sp. and *S. dubia* mitogenomes were assembled as circular molecules of 46,904 bp (GenBank MN642087) and 78,958 bp (GenBank MN642088), respectively. Despite their important variation in size, their gene repertoires are almost identical, with 60 and 61 conserved genes, respectively (duplicated genes were counted only once). The *Tetraselmis* sp. mitogenome is lacking three tRNA genes found in *S*. *dubia* (*trnC*(gcc), *R*(acg), and *T*(ggu)), while the *S*. *dubia* mitogenome is lacking two ribosomal protein-coding genes found in *Tetraselmis* sp. (*rps7* and *rpl10*). Gene order is highly rearranged between the two algal mitogenomes.

Each chlorodendrophycean genome harbors an inverted repeat (IR) but these IRs differ substantially in size and gene content. The 2445-bp IR of *Tetraselmis* sp. contains a single gene (*trnMf*(cau)), whereas the 14,105-bp IR of *S*. *dubia* contains eight genes (including all three rRNA genes) as well as most of the *rps19* coding sequence. Considering that an IR has also been uncovered in the mitogenomes of five prasinophytes representing three distinct classes (Mamiellophyceae, Mamiellales (Robbens et al. 2007; Worden et al. 2009); Palmophyllophyceae, Prasinococcales (Pombert et al. 2013); Pyramimonadophyceae, Pyramimonadales (Satjarak et al. 2017)), it is likely that the IRs reported here represent ancestral features.

The *Tetraselmis* sp. and *S*. *dubia* mitogenomes comprise two and five group I introns, respectively. Both *Tetraselmis* sp. introns reside in *rnl* and encode a LAGLIDADG homing endonuclease, whereas the *S*. *dubia* introns lie within *rns*, *rnl* (two sites), and *cox1* (two sites) and exhibit no ORF. One of the *rnl* introns is inserted at the same site in the two chlorodendrophycean algae.

A maximum-likelihood phylogeny was inferred from 32 concatenated mitogenome-encoded proteins of 17 chlorophytes and 9 streptophyte green algae using RAxML v.8.2.3 (Stamatakis 2014). The best-scoring tree shows that the two representatives of the Chlorodendrophyceae form a strongly supported clade occupying a basal position within the core Chlorophyta (Figure 1).

**Figure 1.**
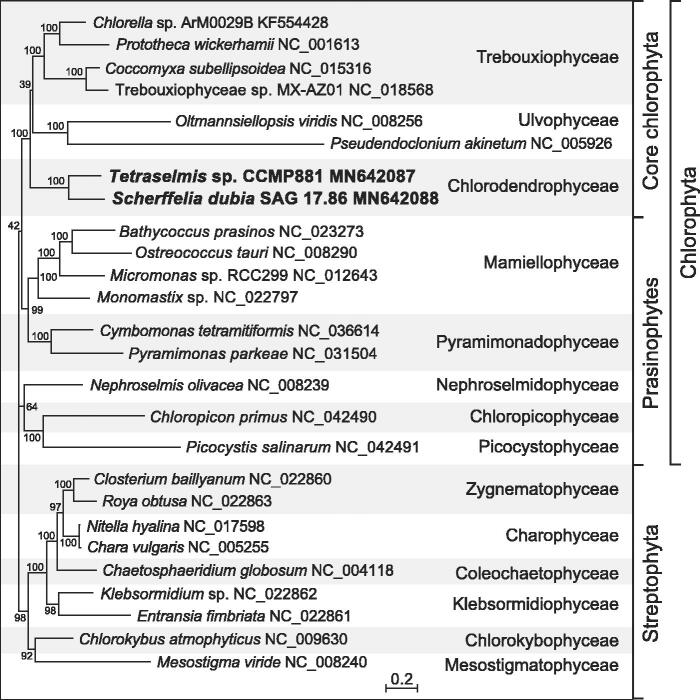
RAxML analysis of 32 concatenated mitogenome-encoded proteins from 17 chlorophytes and 9 streptophyte green algae. The figure shows the best-scoring tree, with the bootstrap support values (100 replicates) reported on the nodes. GenBank accession numbers are provided for the mitogenomes of all taxa. The scale bar denotes the estimated number of amino acid substitutions per site. The data set was generated using the predicted protein sequences derived from the following genes: *atp1*, *4*, *6*, *8*, *9*, *cob*, *cox1*, *2*, *3*, *mttB*, *nad1*, *2*, *3*, *4*, *4L*, *5*, *6*, *7*, *9*, *rpl5*, *6*, *16*, *rps2*, *3*, *4*, *7*, *10*, *11*, *12*, *13*, *14*, and *19*. Following alignment of the sequences of individual proteins with Muscle v3.7 (Edgar 2004), ambiguously aligned regions were removed using TrimAL v1.4 (Capella-Gutierrez et al. 2009) with the options block = 6, gt = 0.7, st = 0.005, and sw = 3, and the protein alignments were concatenated using Phyutility v2.2.6 (Smith and Dunn 2008). For the phylogenetic analysis, the data set was partitioned by protein and the GTR + Γ4 model was applied to each of the 32 partitions.
